# Application and advantages of intrathoracic paravertebral block in uniportal video-assisted thoracoscopic surgery

**DOI:** 10.3389/fsurg.2026.1867293

**Published:** 2026-07-02

**Authors:** Zhengjun Li, Siwei Chao, Shuai Ma, Ding Li, Chang Liu, Sibo You, Guofeng Zhang, Xiangchao Zhang, Shiyuan Song, Tao Wang, Yi Ren

**Affiliations:** 1 Department of Thoracic Surgery, The Tenth People's Hospital of Shenyang, Shenyang, Liaoning, China; 2 Department of Graduate Studies, Shenyang Medical College, Shenyang, Liaoning, China; 3 Department of Anesthesiology, Shenyang 10th People's Hospital, Shenyang, Liaoning Province, China; 4 Department of General Surgery, Operating Room, Shenyang 10th People's Hospital, Shenyang, Liaoning Province, China

**Keywords:** anesthesia, conduction, nerve block, pain, paravertebral block, postoperative, ropivacaine, video-assisted thoracic surgery

## Abstract

**Introduction:**

Uniportal video-assisted thoracoscopic surgery (VATS) is widely used as a minimally invasive approach for pulmonary diseases. Although this technique reduces postoperative pain, acute postoperative pain remains clinically significant. This study aimed to evaluate the feasibility and advantages of thoracoscopic paravertebral block.

**Methods:**

This single-center prospective randomized comparative study included 228 consecutive patients who underwent uniportal VATS at our institution between August 2022 and June 2024. Patients were randomly assigned to Group A (122 patients; intraoperative thoracoscopic direct-vision transthoracic paravertebral block) or Group B (106 patients; preoperative ultrasound-guided thoracic paravertebral block). Perioperative data, postoperative pain scores, opioid rescue analgesic use, and postoperative complications were analyzed. Repeated-measures analysis of variance was used to assess differences in postoperative pain scores over time between the two groups.

**Results:**

A total of 228 patients underwent uniportal VATS pulmonary resection. No significant differences were observed between the two groups in terms of age, sex, pulmonary function, arterial blood gas analysis, surgical side, incision location, nodule size, nodule position, operative time, blood loss, drainage duration, length of hospital stay, tumor stage, or postoperative complications (*P* > 0.05). Postoperative complications were less frequent in Group A than in Group B. No intraoperative or 30-day postoperative mortality occurred in either group. Repeated-measures ANOVA showed significant effects of group (F = 774.002, *P* < 0.001), time (F = 520.972, *P* < 0.001), and group×time interaction (F = 4.983, *P* = 0.001) on VAS scores.

**Conclusions:**

These findings indicate that thoracoscopic paravertebral block is safe and effective. Compared with ultrasound-guided approaches, it was associated with lower postoperative pain scores and reduced rescue tramadol requirements. Further validation through prospective randomized controlled trials is required.

## 1 Introduction

Uniportal video-assisted thoracoscopic surgery (VATS) has become an important minimally invasive approach for pulmonary resection, offering reduced tissue trauma, less postoperative pain, and lower opioid consumption compared with multiportal VATS, while maintaining comparable perioperative and long-term oncological outcomes in lung cancer surgery (1, 2). However, postoperative pain remains a clinically relevant issue after VATS and may result from intercostal nerve compression, chest wall traction, pleural irritation, and thoracic drainage tubes. Inadequate analgesia can impair respiratory function, delay mobilization, reduce patient satisfaction, and contribute to chronic postsurgical pain after VATS pulmonary resection (3, 4).

Current recommendations emphasize multimodal, opioid-sparing analgesia for VATS, including regional anesthesia when appropriate (5). Thoracic paravertebral block (PVB) provides unilateral somatic and sympathetic blockade and has shown effective analgesia after VATS (6, 7). Compared with thoracic epidural analgesia, PVB may provide similar pain control with fewer adverse effects, such as hypotension, urinary retention, and bilateral sympathetic or motor blockade (8).

Although ultrasound-guided thoracic PVB is widely used, its efficacy depends on operator experience and may be limited by anatomical variation, poor acoustic windows, and incomplete visualization of needle placement or local anesthetic spread. Thoracoscopic direct-vision transthoracic PVB allows intraoperative visualization of the paravertebral region and has been reported to be noninferior to ultrasound-guided PVB and feasible after VATS lobectomy (9, 10). However, evidence comparing these two techniques in uniportal VATS remains limited. Therefore, this study aimed to evaluate the feasibility, safety, and analgesic advantages of intraoperative thoracoscopic direct-vision transthoracic PVB compared with preoperative ultrasound-guided PVB in patients undergoing uniportal VATS.

## 2 Materials and methods

### 2.1 Data collection and follow-up

This study analyzed prospectively collected perioperative data from consecutive patients who underwent uniportal VATS at our center between August 2022 and June 2024. Perioperative characteristics and outcome data were reviewed according to a predefined protocol. The inclusion criteria were as follows: American Society of Anesthesiologists physical status grade I–II (11); completion of Visual Analogue Scale (VAS) scoring (12); and absence of surgical contraindications. The exclusion criteria included a history of ipsilateral thoracotomy, prior chest radiotherapy, a history of anesthetic drug allergy, and persistently elevated international normalized ratio and activated partial thromboplastin time on preoperative blood tests. After providing written informed consent, patients were randomly assigned preoperatively to Group A or Group B using a computer-generated random number table. Two surgeons performed all procedures using standardized techniques.

#### 2.1.1 Sample size estimation

Before patient enrollment, the sample size was estimated based on the expected between-group difference in postoperative pain scores between the two paravertebral block techniques. The VAS score at 24 h postoperatively was selected as the primary endpoint for sample size estimation. We assumed a clinically meaningful between-group difference of 1.5 points on the 10-point VAS, corresponding to 15 mm on a 100-mm VAS and exceeding the reported minimal clinically important difference of approximately 10 mm for acute postoperative pain (13). A pooled standard deviation of 2.0 points, a two-sided significance level of 0.05, and a statistical power of 80% were used. Based on a two-sample t-test, at least 29 patients were required in each group. After allowing for an anticipated 10% loss to follow-up, the minimum required sample size was 33 patients per group, corresponding to 66 patients in total. During the study period, all consecutive eligible patients were included, and the final analysis comprised 122 patients in Group A and 106 patients in Group B, which exceeded the minimum required sample size.

### 2.2 Surgical technique

All patients underwent general anesthesia via double-lumen endotracheal intubation to facilitate one-lung ventilation throughout the surgical procedure. The operative approach utilized an array of specialized instrumentation, including long curved endoscopic devices equipped with curved suction attachments and double-jointed mechanical structures, alongside a 10 mm 30-degree angled thoracoscope for optimal visualization. Pulmonary resection and parenchymal transection were performed using the Echelon Flex 30 or 45 articulating endoscopic linear cutting stapler manufactured by Ethicon Endo-Surgery, USA. Each patient was carefully positioned in the full lateral decubitus posture with the operative side facing upward. A solitary surgical incision measuring approximately 3 cm in length was strategically placed at either the fourth or fifth intercostal space along the anterior axillary line. The primary surgeon maintained a consistent position on the ventral aspect of the patient throughout the entire operation. Tissue dissection was accomplished using an ultrasonic scalpel in combination with various other advanced energy-based surgical devices to meticulously divide vascular structures and surrounding soft tissues. Smaller caliber pulmonary vascular branches were securely managed through the application of either metallic vascular clips or conventional silk ligatures depending upon anatomical suitability. Following completion of the resection, all excised pulmonary tissue specimens were carefully retrieved using a protective specimen retrieval bag to prevent wound contamination and potential tumor seeding.For intraoperative and postoperative analgesia, paravertebral blockade was performed using either intraoperative thoracoscopic direct vision or preoperative ultrasound guidance, according to group allocation.Upon completion of the procedure, a thoracic drainage catheter was routinely inserted and connected to a closed suction drainage system. A routine postoperative chest radiograph was obtained on the first postoperative day to comprehensively assess the degree of pulmonary re-expansion and to identify any residual intrathoracic air or fluid collections. The thoracic drainage tube was subsequently removed only when strict criteria were met: the total drainage volume remained below 200 mL over a continuous 24-hour monitoring period, combined with definitive confirmation of absent air leakage characterized by no visible bubbling and complete absence of water column fluctuation during vigorous coughing maneuvers.

### 2.3 Paravertebral block

Patients were divided into two groups according to the approach to PVB: an intraoperative thoracoscopic direct-vision transthoracic PVB group (Group A) and a preoperative ultrasound-guided percutaneous thoracic PVB group (Group B). The thoracic paravertebral space is generally described as a wedge-shaped space bounded anterolaterally by the parietal pleura and posteromedially by the transverse processes and vertebral bodies.

#### 2.3.1 Ultrasound-guided percutaneous thoracic paravertebral block

**Figure 1:**
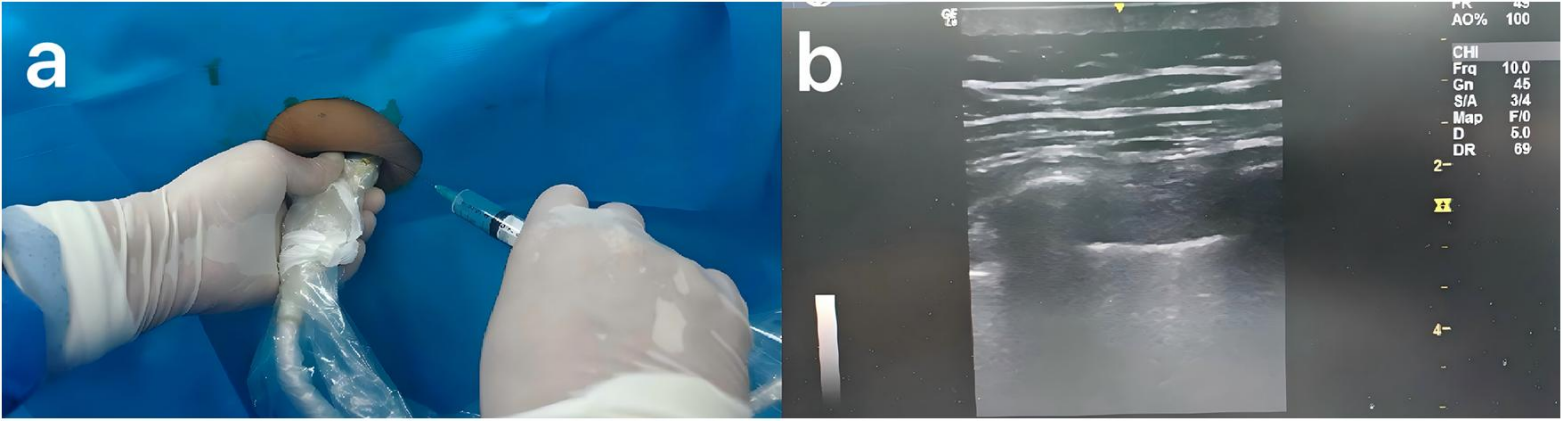
Performance of preoperative ultrasound-guided thoracic paravertebral block. **(a)** Probe placement and needle insertion during ultrasound-guided percutaneous thoracic paravertebral block. The T4–T6 paravertebral spaces were identified approximately 2–3 cm lateral to the spinous processes before uniportal video-assisted thoracoscopic surgery. **(b)** Ultrasound visualization of the thoracic paravertebral space. After negative aspiration, 20 mL of 0.375% ropivacaine was injected, and fusiform spread of the local anesthetic between the pleura and the costotransverse ligament confirmed appropriate distribution within the paravertebral space.

An experienced anesthesiologist with expertise in ultrasound-guided nerve blocks identified the T4–T6 paravertebral spaces under ultrasound guidance, approximately 2–3 cm lateral to the spinous processes. The needle was advanced through the skin, subcutaneous tissue, deep fascia, and erector spinae muscle to reach the paravertebral space. After confirming negative aspiration for blood, 20 mL of 0.375% ropivacaine was injected (Figure 1). Drug diffusion was visualized to confirm that the local anesthetic formed a fusiform spread between the pleura and the costotransverse ligament.

#### 2.3.2 Intraoperative thoracoscopic direct-vision transthoracic paravertebral block

**Figure 2:**
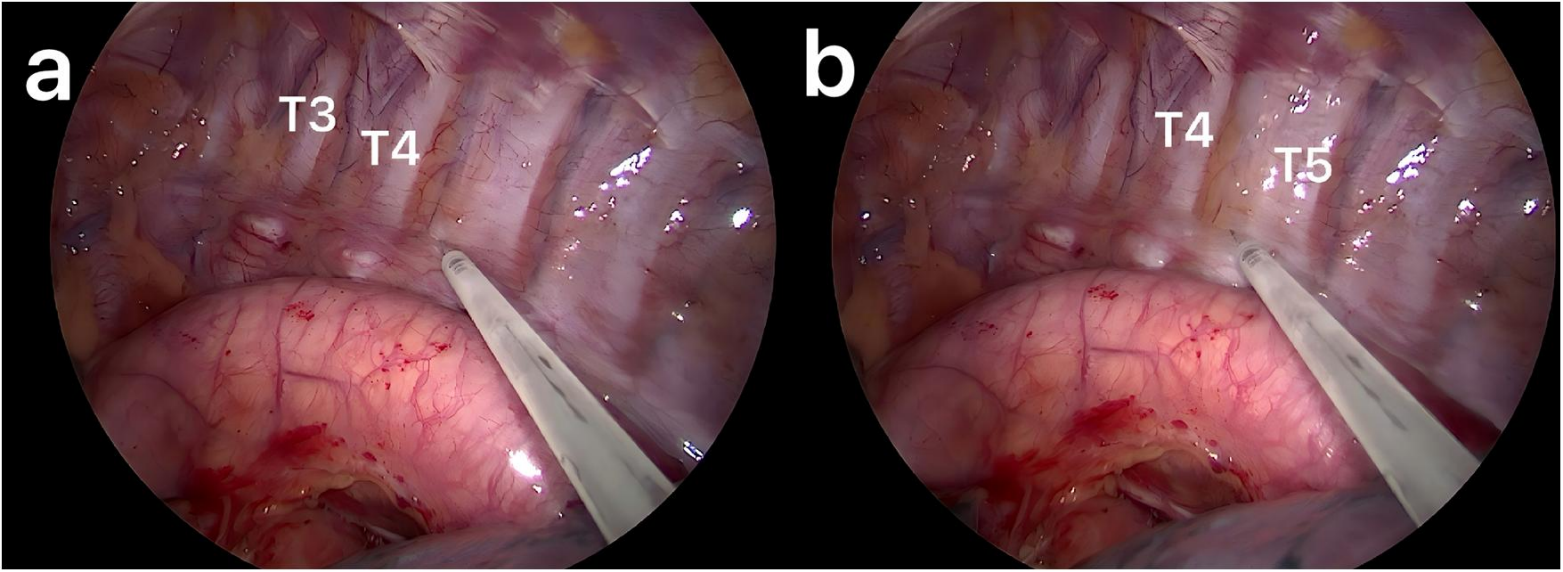
Intraoperative thoracoscopic direct-vision transthoracic paravertebral block. **(a)** Before chest closure, the T4–T6 paravertebral spaces and sympathetic chain were identified under direct thoracoscopic visualization. The block needle was inserted along the upper border of the rib and advanced 0.5–1 cm to enter the paravertebral space. **(b)** After negative aspiration, 20 mL of 0.375% ropivacaine was injected. The visible subpleural elevation indicated successful spread of the local anesthetic within the thoracic paravertebral space beneath the parietal pleura, supporting effective postoperative analgesia.

Before chest closure, a trained surgeon identified the T4–T6 paravertebral spaces under thoracoscopic direct vision. The sympathetic chain was visualized thoracoscopically. The needle was inserted approximately 0.5 cm lateral to the distal end along the upper border of the rib and advanced 0.5–1 cm to enter the paravertebral space (Figure 2). After confirming negative aspiration for blood, 20 mL of 0.375% ropivacaine was injected.

### 2.4 Pain management

Both groups received a standardized multimodal postoperative analgesic regimen. Postoperative analgesia was administered by nurses specialized in pain management according to a predefined protocol. All patients received intravenous ketorolac tromethamine 30 mg twice daily and acetaminophen every 6 h after surgery. If patients reported severe postoperative pain, defined as a VAS score ≥ 4 despite routine analgesia, tramadol was administered as rescue analgesia at a fixed dose of 100 mg intravenously every 12 h as needed. The number of tramadol administrations was recorded in the nursing documentation. No patient received more than two administrations of rescue tramadol during the postoperative observation period.

### 2.5 Visual analogue scale pain scoring

Postoperative pain intensity was assessed using the VAS, a 10-point scale ranging from 0 to 10, where 0 indicated no pain and 10 indicated the most severe unbearable pain (12). Patients were asked to indicate the score that best represented their pain intensity at each assessment time point. Because of the different timing and technical approaches of the two paravertebral block procedures, blinding of procedural operators and participants was not feasible. To reduce assessment bias, postoperative VAS scores were recorded by trained pain-management nurses who were not involved in the paravertebral block procedures and were instructed not to review group allocation. VAS scores were recorded at 6, 12, 24, 48, and 72 h postoperatively.

### 2.6 Statistical analysis

Categorical variables were expressed as number and percentage, and continuous variables were expressed as mean ± standard deviation. The normality of continuous variables was assessed before analysis. Group comparisons were performed using Student's t-test for normally distributed continuous variables. Categorical variables were compared using the chi-square test or Fisher's exact test, as appropriate. Fisher's exact test was used when the expected cell count was small. Differences in VAS scores between the two groups over time were analyzed using repeated-measures analysis of variance. Mauchly's test was used to assess the assumption of sphericity; when the assumption was met, uncorrected repeated-measures ANOVA results were reported. Data analysis was performed using IBM SPSS Statistics for Windows, version 26.0 (IBM Corp., Armonk, NY, USA). Statistical significance was set at *P* < 0.05 using a two-tailed test. Graphs were generated using GraphPad Prism, version 8.3.1.

## 3 Results

### 3.1 Patient characteristics

Between August 2022 and June 2024, a total of 228 patients underwent uniportal VATS lung resection. No intraoperative or 30-day postoperative mortality was observed. Among these patients, 122 were assigned to Group A and 106 to Group B. Patient characteristics are summarized in Table 1. No statistically significant differences were found between the two groups in terms of age, sex, arterial blood gas analysis, laterality, pulmonary function, incision location,nodule position, nodule size, or surgical approach (*P* > 0.05).

**Table 1:** Baseline patient characteristics in group A and group B.

Characteristic	Group A(*n* = 122)	Group B(*n* = 106)	Total(*n* = 228)	*P* value
Age (years), n(%)				0.053
≤60(%)	82 (67.21)	57 (53.77)	139 (60.96%)	
>60(%)	40 (32.79)	49 (46.23)	89 (39.04%)	
Mean ± SD	57.03 ± 8.98	58.05 ± 9.55	57.50 ± 9.24	0.410
Gender, *n* (%)				0.746
Male	52 (42.62)	42 (39.62)	94 (41.23%)	
Female	70 (57.38)	64 (60.38)	134 (58.77%)	
ASA grade, *n* (%)				
I	38 (31.15)	47 (44.34)	85 (37.28%)	0.055
II	84 (68.85)	59 (55.66)	143 (62.72%)	0.097
Pulmonary function (Mean ± SD)				
FEV1	93.34 ± 16.96	92.67 ± 16.16	93.03 ± 16.56	0.763
Laterality, *n* (%)				0.961
Left	52 (42.62)	39 (36.79)	91 (39.91%)	
Right	70 (57.38)	67 (63.21)	137 (60.09%)	
Incision location, *n* (%)				0.423
4th intercostal space	66 (54.10)	44 (41.51)	110 (48.25%)	
5th intercostal space	56 (45.90)	62 (58.49)	118 (51.75%)	
Operation method, *n* (%)				0.971
Wedge resection	31 (25.41)	28 (26.42)	59 (25.88%)	
Segmentectomy	12 (9.84)	11 (10.38)	23 (10.09%)	
Lobectomy	79 (64.75)	67 (63.21)	146 (64.04%)	

Data are presented as number (%) or mean ± standard deviation (SD).

Comparisons between groups were performed using the chi-square test for categorical variables and the independent-samples t-test for continuous variables.

ASA, American Society of Anesthesiologists; FEV1, forced expiratory volume in 1 s. *P* values <0.05 were considered statistically significant.

### 3.2 Surgical outcomes

No significant differences were observed between the two groups in operative time, blood loss, drainage duration, length of hospital stay, or tumor stage (*P* > 0.05). Postoperative pathological diagnoses included benign lesions and primary lung cancer, with no significant difference between the groups (*P* > 0.05). Postoperative complications occurred in 1 patient in Group A and 9 patients in Group B, corresponding to complication rates of 0.8% and 8.5%, respectively. The difference between the two groups was not statistically significant (*P* = 0.404; Table 2). No intraoperative or 30-day postoperative deaths occurred in either group. No significant difference was observed in the proportion of patients who experienced agitation after tracheal extubation.

**Table 2:** Perioperative outcomes in Group A and Group B.

Characteristic	Group A (*n* = 122)	Group B (*n* = 106)	Total (*n* = 228)	*P* value
Surgical results(Mean ± SD)				
Operative time (min)	145.75 ± 38.96	136.85 ± 35.55	141.61 ± 37.59	0.075
Blood loss (mL)	51.89 ± 26.95	48.16 ± 28.73	50.15 ± 27.79	0.314
Drainage time (days)	5.51 ± 1.97	5.25 ± 2.02	5.39 ± 2.00	0.329
Length of hospital stay (days)	7.25 ± 2.97	6.89 ± 3.26	7.08 ± 3.11	0.385
Postoperative complications, *n* (%)				
Total	1 (0.82)	9 (8.49)	10 (4.39)	0.404

Data are presented as mean ± standard deviation for continuous variables and number (%) for categorical variables.

Comparisons between groups were performed using the independent-samples t-test for continuous variables and the chi-square test or Fisher's exact test for categorical variables, as appropriate.

Fisher's exact test was used for postoperative complications because of the small number of events.

*P* values <0.05 were considered statistically significant.

### 3.3 Postoperative opioid use

Among all patients, 54 patients (23.68%) experienced severe postoperative pain and received rescue tramadol. Rescue tramadol was required in 15 patients in Group A and 39 patients in Group B (*P* < 0.001). A single administration of tramadol was required in 9 patients in Group A and 31 patients in Group B (*P* < 0.001), whereas two administrations were required in 6 patients in Group A and 8 patients in Group B (*P* = 0.584). No patient in either group required more than two administrations of rescue tramadol. These findings indicate that thoracoscopic direct-vision transthoracic PVB was associated with reduced postoperative rescue opioid requirements compared with ultrasound-guided PVB.

### 3.4 Visual analogue scale pain scores

Postoperative pain was assessed using the VAS. The Shapiro–Wilk test confirmed that VAS scores were normally distributed in both groups. Mauchly's test of sphericity showed *W* = 0.953 and *P* = 0.298, indicating that the sphericity assumption was met; therefore, standard repeated-measures ANOVA was performed without correction. Mean VAS scores in Group A vs. Group B were 3.46 ± 0.56 vs. 4.61 ± 0.52 at 6 h, 2.83 ± 0.53 vs. 3.71 ± 0.60 at 12 h, 2.18 ± 0.68 vs. 3.29 ± 0.66 at 24 h, 1.70 ± 0.52 vs. 2.66 ± 0.61 at 48 h, and 1.39 ± 0.61 vs. 2.11 ± 0.60 at 72 h, respectively. Repeated-measures ANOVA demonstrated significant effects of group, time, and group×time interaction on VAS scores (*P* < 0.05). Specifically, significant effects were observed for group (F = 774.002, *P* < 0.001), time (*F* = 520.972, *P* < 0.001), and group×time interaction (*F* = 4.983, *P* = 0.001; Table 3).

**Table 3:** Comparison of visual analogue scale (VAS) scores between Group A and Group B at different postoperative time points.

Group	VAS scores					Repeated measures *F* test	
	6h	12 h	24h	48 h	72 h	F	*P* value
Group A	3.46 ± 0.56	2.83 ± 0.53	2.18 ± 0.68	1.70 ± 0.52	1.39 ± 0.61		
Group B	4.61 ± 0.52	3.71 ± 0.60	3.29 ± 0.66	2.66 ± 0.61	2.11 ± 0.60		
Group effects						774.002	<0.001
Time effects						520.972	<0.001
Group × Time effects						4.983	0.001
Normally distributed data compared using repeated measures ANOVA(*P* < 0.05)							

Data are presented as mean ± standard deviation (SD).

Normally distributed data were analyzed using repeated measures analysis of variance (ANOVA) to assess the effects of group, time, and group×time interaction.

Significant differences were observed between groups, across time points, and in the interaction effect (*P* < 0.05).

Simple-effects analysis showed significant differences in VAS scores between the two groups at 6 h (*F* = 254.313, *P* < 0.001), 12 h (*F* = 136.690, *P* < 0.001), 24 h (*F* = 156.310, *P* < 0.001), 48 h (*F* = 164.983, *P* < 0.001), and 72 h (*F* = 80.880, *P* < 0.001). VAS scores decreased progressively over time in both Group A and Group B (both *P* < 0.001). Subgroup analyses stratified by surgical laterality and incision location showed that the analgesic advantage of Group A over Group B remained significant across different operative sides and incision locations (Table 4).

**Table 4:** Postoperative VAS scores stratified by surgical laterality and incision location in Group A and Group B.

VAS scores (Mean ± SD)	Group A (*n* = 122)	Group B (*n* = 106)	Total (*n* = 228)	*P* value
Laterality (Left vs Right)				
6 h	3.48 ± 0.51	4.65 ± 0.49	3.98 ± 0.50	<0.001
12 h	2.80 ± 0.56	3.87 ± 0.52	3.26 ± 0.54	<0.001
24 h	2.12 ± 0.73	3.18 ± 0.54	2.58 ± 0.66	<0.001
48 h	1.73 ± 0.53	2.69 ± 0.70	2.14 ± 0.61	<0.001
72 h	1.51 ± 0.60	2.09 ± 0.61	1.76 ± 0.60	<0.001
Incision location (4th vs 5th intercostal space)				
6 h	3.44 ± 0.60	4.50 ± 0.54	3.87 ± 0.57	<0.001
12 h	2.86 ± 0.49	3.70 ± 0.56	3.20 ± 0.52	<0.001
24h	2.23 ± 0.71	3.35 ± 0.68	2.68 ± 0.70	<0.001
48 h	1.72 ± 0.55	2.71 ± 0.56	2.12 ± 0.55	<0.001
72 h	1.49 ± 0.61	2.17 ± 0.57	1.77 ± 0.60	<0.001

Data are presented as mean ± standard deviation.

Comparisons between groups were performed at each postoperative time point.

The results showed that Group A had significantly lower VAS scores than Group B at all postoperative time points after stratification by surgical laterality and incision location.

## 4 Discussion

Although VATS is minimally invasive, postoperative pain remains a significant issue. Reported rates of moderate-to-severe postoperative pain after VATS vary widely depending on assessment timing and definitions, ranging from approximately 15% within 48 h to higher proportions in selected cohorts (14). Inadequate analgesia may prolong discomfort and increase the risk of pulmonary complications, thereby limiting recovery. In the context of enhanced recovery after surgery protocols, optimizing perioperative analgesic strategies for VATS patients has become a clinical priority (15).

Epidural anesthesia was initially advocated for effective pain management following thoracic surgery; however, it is associated with a relatively high incidence of side effects and potential complications. In recent years, regional anesthesia techniques have been increasingly adopted in clinical practice because they reduce postoperative pain and opioid consumption. PVB has gained increasing recognition as a regional analgesic technique for thoracic procedures (16). Its mechanism of action involves direct blockade of afferent pain pathways, which reduces central sensitization and controls pain at its source.

Compared with intravenous opioid analgesia or epidural analgesia, PVB more effectively attenuates surgical stress responses, reduces perioperative opioid consumption, improves pulmonary function impaired by incisional pain, and has minimal hemodynamic impact. These advantages support its role as a key component of enhanced recovery after surgery protocols. An updated meta-analysis by Ding et al., which included 18 randomized trials, reported comparable pain scores between PVB and epidural analgesia at 4–8, 24, and 48 h (mean difference 0.06; 95% confidence interval −0.31 to 0.42; *P* = 0.77), with significantly lower rates of urinary retention, nausea and vomiting, and hypotension with PVB (17).

The successful implementation of thoracic PVB depends on precise anatomical targeting of the paravertebral space. This region is bounded anterolaterally by the parietal pleura and contains branching spinal nerves accompanied by abundant vasculature, requiring accurate nerve localization and careful avoidance of blood vessels and the pleura during needle placement. The ultrasound-guided approach improves success rates; however, its tomographic imaging characteristics and depth limitations may result in suboptimal needle tip visualization and inaccurate assessment of drug diffusion, particularly in patients with obesity, pleural effusion, or anatomical variation. These limitations may lead to incomplete blockade or complications such as vascular injury or pneumothorax (18).

In recent years, thoracoscopic-assisted transthoracic PVB has emerged as a promising solution to these challenges. By leveraging real-time direct visualization, thoracoscopy allows surgeons to clearly identify nerve trajectories, vascular structures, and pleural reflections within the paravertebral region, enabling precise, real-time visually guided interventions. The results of this study suggest that, compared with conventional ultrasound-guided percutaneous puncture, the thoracoscopic direct-vision approach may enhance procedural visualization, improve anatomical accuracy during puncture, and provide more reliable visualization of the needle trajectory and local anesthetic spread. These findings support that the direct-vision approach may reduce the risk of collateral damage, including sympathetic nerve injury.

The thoracoscopic direct-vision approach demonstrates particular value in patients with suboptimal ultrasound imaging. It provides clear visualization of the needle trajectory and enables real-time assessment of local anesthetic diffusion, thereby ensuring block efficacy and improving postoperative analgesic quality. However, this technique is typically performed in conjunction with thoracoscopic surgery, involves additional invasiveness, and is not suitable as a standalone analgesic method in non-surgical patients. Although rare complications such as inadvertent intrathecal injection and total spinal anesthesia have been reported with thoracic PVB (19), no such events occurred in this study.

This study has several limitations. First, this was a single-center study, and the findings may not be fully generalizable to other institutions with different surgical or anesthetic practices. Second, although the final sample size exceeded the prespecified minimum requirement, the sample size remained relatively limited, particularly for the assessment of uncommon complications. Third, because of the distinct timing and technical characteristics of thoracoscopic direct-vision PVB and ultrasound-guided PVB, blinding of the procedural operators and participants was not feasible, which may have introduced performance or perception bias. To reduce assessment bias, VAS scores were recorded by trained pain-management nurses who were not involved in the block procedures. Fourth, this study focused primarily on short-term postoperative outcomes, and long-term outcomes such as chronic postsurgical pain, long-term quality of life, and persistent opioid use were not evaluated. Finally, the effectiveness of both techniques may be influenced by operator experience, and further multicenter randomized studies with longer follow-up are needed to validate these findings.

In summary, during thoracoscopic lung resection, thoracoscopic-direct vision PVB offers high precision and safety and serves as an effective complementary technique. This approach may facilitate the development of more individualized and reliable strategies for postoperative analgesia in thoracic surgery.

## Data Availability

The raw data supporting the conclusions of this article will be made available by the authors, without undue reservation.
